# On-chip multivariant COVID 19 photonic sensor based on silicon nitride double-microring resonators

**DOI:** 10.1515/nanoph-2022-0722

**Published:** 2023-03-31

**Authors:** Arieh Grosman, Tal Duanis-Assaf, Noa Mazurski, Roy Zektzer, Christian Frydendahl, Liron Stern, Meital Reches, Uriel Levy

**Affiliations:** Department of Applied Physics, The Benin School of Engineering and Computer Science, The Hebrew University of Jerusalem, 91904, Jerusalem, Israel; The Institute of Chemistry, The Hebrew University of Jerusalem, 91904, Jerusalem, Israel; The Center for Nanoscience and Nanotechnology, The Hebrew University of Jerusalem, 91904, Jerusalem, Israel

**Keywords:** biosensors, COVID-19, micro ring resonator; nanophotonics; silicon-photonics; Si_3_N_4_

## Abstract

Coronavirus disease 2019 (COVID-19) is a newly emerging human infectious disease that continues to develop new variants. A crucial step in the quest to reduce the infection is the development of rapid and reliable virus detectors. Here, we report a chip scale photonic sensing device consisting of a silicon-nitride double microring resonator (MRR) for detecting SARS-CoV-2 in clinical samples. The sensor is implemented by surface activation of one of the MRRs, acting as a probe, with DNA primers for SARS-CoV-2 RNA, whereas the other MRR is used as a reference. The performance of the sensor is determined by applying different amounts of SARS-CoV-2 complementary RNA. As will be shown in the paper, our device detects the RNA fragments at concentrations of 10 cp/μL and with sensitivity of 750 nm/RIU. As such, it shows a promise toward the implementation of label-free, small form factor, CMOS compatible biosensor for SARS-CoV-2, which is also environment, temperature, and pressure independent. Our approach can also be used for detecting other SARS-CoV-2 genes, as well as other viruses and pathogens.

## 1 Introduction

The 2019 global COVID-19 pandemic pushed many governments to enforce disease containment measures and prevent further spreading. This includes quarantine and isolation of infected individuals, as well as case contact tracing [1]. While these seem to be effective measures [2], a fast and reliable diagnosis methodology is required to effectively track and isolate patients [3].

To this day, polymerase chain reaction (PCR) remains the most widely used method for COVID-19 diagnosis [4, 5]. However, the need for specialized equipment and personnel as well as the lack of availability of mass quantities of PCR kits are a concern. In some cases, laboratories resort to test pooling, to reduce the workload and deliver faster results [6]. Therefore, there is a growing interest in alternative testing methods. A rapid bio detector capable of detecting a minimal amount of the virus is a crucial stage for this mission. For label-free detectors, the limit of detection (LOD) is typically defined as the minimum refractive index unit (RIU) change necessary to cause a detectable change in the output signal [7]. The LOD can also be defined by the minimal detectable concentration, which is typically measured in viral concentration for detectors, which target the virion particle, or in copies of the target gene per volume (cp/μL) for detectors, which target genetic material [8]. A PCR test has a LOD of 8.31 × 10^1^ cp/mL for sputum test during 3 h of processing time [9]. Moitra and coworkers [10] have developed a test to diagnose COVID-19 that can detect the virus in 10 min using the advantage of plasmonic gold nanoparticles, and the limit of detection was found to be 0.18 ng/μL. G. Seo et al. report graphene sheets of the FET with a LOD of 1 fg/mL and clinical samples LOD of 2.42 × 10^2^ cp/mL [11]. Recently, a COVID-19 sensor based on interferometric bimodal waveguides (BiMWs) [12] was demonstrated, with a limit of detection of 10^−8^ RIU and clinical samples LOD 10^2^–10^3^ viruses/mL. For MRR-based biosensors, MS McClellan reported an MRR sensor for *Bean pod mottle virus*, with a limit of detection of 10 ng/mL within 45 min incubation time [13] and 10^−7^ RIU resolution [7, 14, 15].

Microresonators, such as microsphere resonators, microbubble resonators, and microtoroid resonators, can be implemented with high Q factors. This, combined with the sensitivity of the wavelength of resonance to small changes in the refractive index of the surrounding, enabled the application of such resonators as biochemical sensors down to the single molecule level [16–21]. To further enhance the integration levels, such sensors can be implemented as MRRs. And indeed, MRRs based on silicon, silicon oxide, and silicon nitride microring resonators (MRRs) are micro/nanoscale structures composed of waveguides coupled to microrings. These microresonators typically provide high sensitivity, fast readout (real-time), small footprint, and compatibility with complementary metal-oxide semiconductor (CMOS). These merits make the MRR attractive for wide range of applications such as light sources [22, 23], modulators [24–26], and sensors [27–30], to name a few.

Due to their high sensitivity to small changes in refractive index, MRRs can be used to detect very small changes on the MRR surface. Based on this property, MRRs were used for label-free detection of DNA [14], viruses [13], and RNA. Recently, we have introduced the concept of the double MRR platform and demonstrated its ability to measure ultra-low optical signals [31]. This double MRR platform not only provides a reference on the same chip to eliminate environmental effects such as temperature fluctuations but also allows to measure frequency differences in the RF domain, very similar to implementation of frequency standards and atomic clocks. As such, this platform is ideal for the development of biosensors designed to detect biological entities in highly diluted solutions.

In this work, we demonstrate the design, fabrication, and experimental characterization of a bio-sensor platform consisting of double MRR structure made of standard CMOS compatible materials. Specifically, Si_3_N_4_ is used as the waveguide core, whereas SiO_2_ is used as a substrate and as cladding. The two MRRs provide both the reference and the probe and are exposed from the top by removing the SiO_2_ cladding. The device was designed to sense SARS-CoV-2 in different patient samples, such as nasopharyngeal, nasal, or saliva by chemically activating the probe MRR surface with a primer targeting viral RNA. Based on the demonstrated devices, our device was able to detect RNA fragments at concentrations down to the ∼10 cp/μL level and with sensitivity of 743 nm/RIU. These results demonstrate that MRR sensors can serve as a new promising clinical diagnostics technology. It is worth mentioning that the target RNA tested here is a synthetic 880-base nucleic acid chain, whereas the full SARS-COV-2 genome is approximately thirty thousand nucleobases long [32]. Therefore, with the natural genetic material, the device sensitivity will likely be even higher. On that note, our setup should be further studied using clinically relevant samples, such as nasal swabs, to evaluate its performance in the presence of biological “contaminants” (proteins and DNA), before clinical studies can commence. Nevertheless, the high specificity of nucleic acids makes recognition of nontarget molecules improbable. Thus, only nonspecific adhesion of biological molecules to the MRR surface can lead to false detection. However, both the reference and probe MRRs are subjected to the same nonspecific adhesion, and therefore, the use of the two MRR setup absolves the issue of nonspecific adhesion.

## 2 Results and discussion

Resonators are an excellent choice to track small changes in the refractive index by monitoring the spectral shift of the resonance wavelength. The resonance condition is obtained when the effective length of the MRR (its perimeter multiplied by the effective index of the mode) is an integer multiplication of the resonance wavelength. We can thus express the resonance condition as [14, 15] $\lambda_{\text{res}}=\frac{n_{\text{eff}}L}{m}$ , where *n*_eff_ is the effective refractive index of the $Si_{3}N_{4}\left(SIN\right)$ core waveguide. For biosensing applications, the waveguides should operate in a single-mode regime, to avoid ambiguity due to overlapping resonances of higher order modes. The cross-sectional dimensions of the *SIN* waveguide core were chosen to be 400 nm height and 1 μm width. The MRR radius was set to 200 μm. The waveguide dimensions support a TE mode at wavelengths around 1550 nm. This mode was used in our experiment. Basically, our design supports also TM mode, which could experience less scattering loss from sidewalls because the mode is guided above and below the waveguide (resulting from fabrication). On the other hand, the bending loss is higher for TM mode and a larger MRR radius is needed in order to maintain a high Q factor [33, 34].

**Figure 1:**
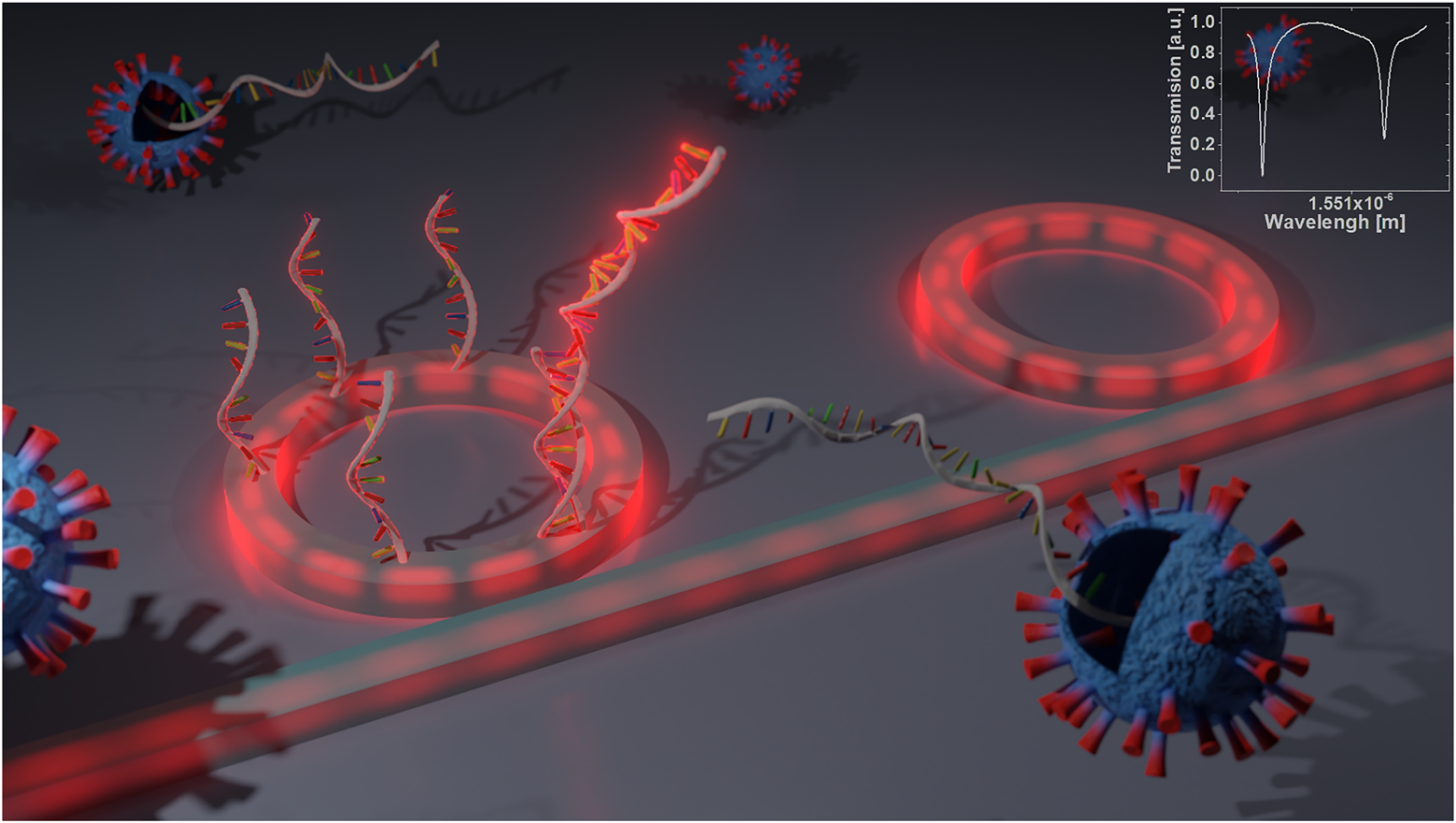
A double MRR structure consisting of two identical MRRS (200 μm radius), where light is coupled to each of the MRRs through a 1 μm width ridge waveguide, separated by ∼500 nm gap from the MRRs. The advantage of a double MRR configuration is the ability to eliminate environmental changes such as small changes in temperature. One of the MRRS is exposed to a surface activation process, where a PCR primer is attached to the MRR. Inset: Spectral resonances of the double MRR system.

The MRR acts as a label-free biosensor that can directly measure selective interactions between analyte molecules and receptor molecules on its surface (Figure 1). The evanescent tail of the optical field interacts with the cladding material and hence the change in refractive index due to the presence of the analyte results in a change in the effective index of the waveguide mode, and correspondingly a shift in the resonance wavelength. For small variations of the upper-cladding refractive index (Δ*n*_*c*_), first-order correction to the resonance wavelength can define the sensitivity by $\frac{\Delta \lambda }{\lambda }=\Gamma \frac{\Delta n}{n_{\text{eff}}}$ , where *λ*_0_ is the resonance wavelength of the unperturbed resonator, *n*_eff_ is the effective index of the waveguide (∼2.04 in our case), and Γ is the proportionality factor, which is related to the sensitivity of the device according to Δ*n*_eff_ = ΓΔ*n*_*c*_.

An important issue to consider is the measurement error, which can lead to a false-positive/negative COVID-19 test. MRRs are ultra-sensitive to minute changes in the effective refractive index [35]. As such, any small fluctuation in the environment temperature or pressure can lead to an undesired shift of the resonance peak wavelength resulting in a measurement error. For instance, the temperature sensitivity of the MRR sensors made of silicon was reported to be ∼70 nm/RIU [15]. Considering the relatively large thermo-optic coefficient of silicon, ∼1.8 × 10^−4^ K^−1^, this implies that excellent temperature stability is required for the operation of the sensor, which in some conditions may be extremely challenging, particularly during the measurement, where light is circulating within the resonator.

In this work, we eliminate this limitation using 3 main methodologies. First, we use a SIN on SiO_2_ substrate as a sensor platform for the bus waveguide and the MRRs. An important advantage of SiN for biosensing applications is its low thermo-optic coefficient (∼2 × 10^−5^ K^−1^) and the lack of two photon absorption mechanism at the operation wavelength, making it less sensitive to environmental variations [35]. Second, and perhaps most importantly, we have used a double MRR system, instead of a single MRR. The use of the double identical MRRs configuration allows the elimination of environmental changes such as small fluctuations in temperature and pressure, operating in a common mode rejection [31]. Third, for measurements control and verification, we performed the measurement during thermal stabilization of the system (Thorlabs TED2000, TEC CUI devices cp3028, Thorlabs ad590), to decrease the error measurement due to thermal fluctuations.

Our device is implemented such that one of its MRRs is used as the probe, while the other serves as a reference. The probe MRR was functionalized with a PCR primer, targeting the coronavirus RNA, while the reference MRR was kept unfunctionalized. During measurement, both MRRs were exposed to the target RNA. The RNA was expected to specifically bind to the primer bearing probe MRR but not to the reference MRR. Thus, the reference MRR serves as an internal negative control, which is only subject to nonspecific adhesion of the analyte.

**Figure 2:**
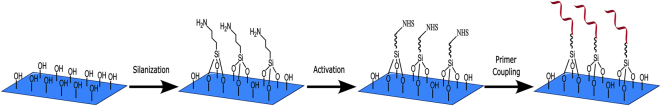
Surface activation chemical scheme.

To facilitate molecular recognition of viral RNA, a PCR primer was chemically bounded to the SiN probe MRR. We used a well-known SARS-CoV-2 PCR primer targeting the coronavirus N-gene, designed by the United States centers for disease control and prevention (CDC) [36]. The probe MRR surface activation is described in Figure 2, where, as the initial step, the surface was activated using O_2_ plasma treatment following surface silanization with amine-terminated groups. This was followed by chemical attachment of a short, flexible linker, and chemical activation to allow the attachment of the primer as the last step. The purpose of the flexible linker was to allow the primer to adopt an optimal orientation, leaving it free to interact with the target RNA. The chemical attachment of the linker and subsequent primer coupling reaction were only applied on the probe MRR by means of a physical barrier, which prevented the coupling cocktails from leaking toward the reference MRR. In that sense, only the probe MRR can specifically bind the target RNA, whereas the reference ring is only subject to nonspecific interactions with the tested solution. The surface activations describe in more detail in the Materials and Methods section.

**Figure 3:**
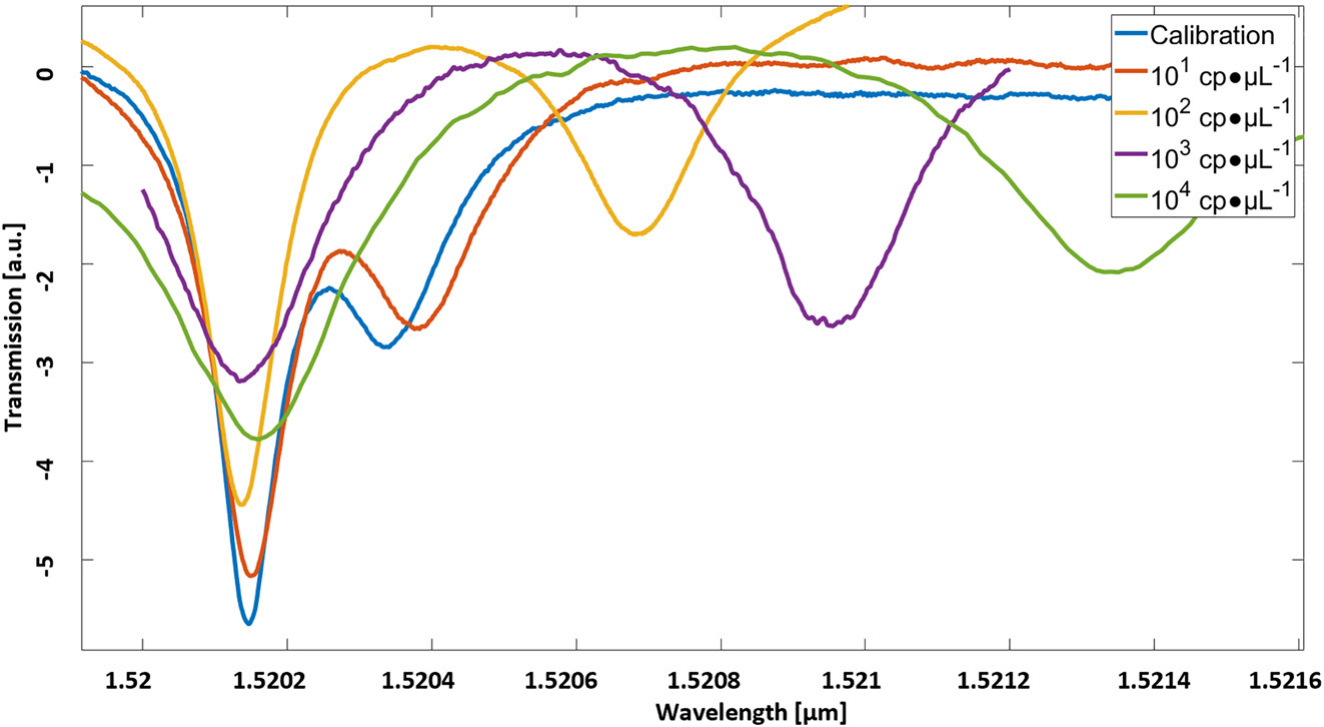
The spectral transmission measurement results of our double MRR device. The spectral separation between the two resonances is increased with the increase of cp of RNA attachment to the primer on the surface of the MRR, because of the increase in the effective refractive index of the mode.

To characterize the fabricated device, we performed transmission measurements using a tunable laser operating at wavelengths around 1550 nm (HP 81608A), where the light is delivered into the chip using a lens fiber and coupled to the chip through an inverse tapered waveguide, using the edge (butt) coupling method. According to the experimental results presented in Figure 3, we obtained a quality factor (Q factor) of ∼10^5^ and extinction ratio up to 15 dB. While higher Q factors can now be achieved, this Q factor is still considered as decent as compared to other works, e.g., [37] and is sufficient to achieve our goals, as will be shown later in the text.

Typical spectra obtained using our device can be seen in Figure 3. The spectra show two Lorentzian-shaped resonances, corresponding to the two MRRs. First, a calibration curve was taken before exposure to the analyte. Next, three other curves were taken after incubation with analyte solution in different concentrations. When comparing the two resonances of the calibration curve to those of the other curves, a distinct resonance spectral shift is detected, and the spectral range between the two resonances (corresponding to the two MRRs) is increased with the increase in analyte concentration.

After incubation, the target RNA is likely to be bounded to the probe MRR via the primer sequence. As a result, the light propagating within the MRR interacts with the RNA molecules on top via the evanescent “tail” of the mode [16]. This leads to a shift in the effective index of the mode and correspondingly a shift in the resonance. Importantly, a sharp spectral shift was only noted for one of the two resonances. This implies the RNA was only bounded to the probe MRR, while the reference MRR was kept unchanged.

**Figure 4:**
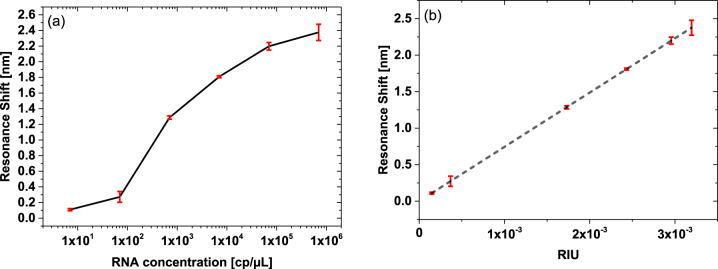
Device measurments. (a) Measured spectral shift between the probe and reference MRRs as a function of the RNA concentration. From the results, we estimate the detection limit to be in the order of ∼10 cp/μL. (b) Measured spectral shift as a function of the change in RIU. The dashed red line is a linear fit to the measured results. The slope corresponds to $743\frac{nm}{RIU}$ .

During the measurements, we examined the resonance shift of both MRRs after exposure to the target RNA. Because it wasn’t chemically functionalized, the reference MRR is not expected to specifically bind the target RNA. Rather, it is only subject to nonspecific interactions with the analyte. This configuration allows us to measure nonspecific RNA attachment (represented in Supplementary Figure S2). In this way, we ensure the shift is only caused by the attached RNA on the probe MRR and not by contamination or nonspecific attachment. The measured transmission spectrum as a function of the analyte RNA concentration can be seen in Figure 4a. From the results, we estimate the detection limit to be in the order of ∼10 cp/μL.

In Figure 4b, one can see the slope of the resonance shift versus the refractive index change (RIU). The latter was extracted from the measured free spectral range (FSR) of the device transmission (full spectral range, not shown). For each of the different concentrations, the FSR was measured, from which the effective index was extracted. This slope of the spectral shift versus RIU defines the sensitivity of the device (according to Δ*λ*/*λ* = Δ*n*/*n*_eff_). The slope of our device was found to be as high as **743 nm/RIU** (applying **Γ** = **1**).

We reproduced each measurement at least 3 times, while stabilizing the temperature to 40 °C. At high concentrations, above 10^4^ cp μL^−1^, a saturation of the spectral shift was observed. The probable reason for this saturation is related to the density of the primer with respect to the concentration of complementary RNA molecules. As this concentration is increased beyond a certain value, most of the primers are becoming attached with complementary RNA. Thus, not enough active sites remain free for binding additional RNA molecules. This behavior is expected if the number of target RNA molecules is significantly higher than the number of active sites on the MRR surface. This limitation could be mitigated by increasing the surface concentration of the primer, e.g., by optimizing the chemical functionalization process. Another possible explanation for the saturation effect is the prevention of RNA adhesion due to physical hindrance, such as steric or coulombic repulsion [38–40]. Such a physical barrier is likely to increase when using the full viral genome, rather than the relatively small RNA fragments used in this study. Nonetheless, this can be solved by adjusting the physical size of the MRR according to the expected RNA amount for the test. A small variation in sensitivity was seen for different devices, as shown in Supplementary Figure S3. This possibly arises from the tolerance in fabrication of the device.

As always, there is some variance between one fabricated device to another, originating from the device fabrication tolerances. The overall tolerances of our device are determined by two major factors: the tolerance of the dimensions of the two (nearly identical) MRRs, which are limited mostly by the precision of the lithography process, and the tolerance in primer density variability on the surface of the probe MRR. The electron beam lithography has a tolerance of a few nanometers, supporting our capability to fabricate two MRRs having two desirable resonances. The spectral separation between these two resonances may shift slightly due to fabrication tolerances. However, this fabrication tolerance has only a minor effect on the sensitivity of the device due to the calibration process. This calibration consists of an initial measurement of the spectral separation between the two resonances, and the shift that is resulting from the additional RNA attached to the surface, as shown in Figure 3, is related to the initial spectral separation. In Supplementary Figure S4, one can see a calibration measurement of 3 different devices with slight variations as an outcome of the lithography tolerance (device #1 – black line, device #2 – red line, device #3 – blue line). As can be seen, the spectral distance (Δ) between the reference and the probe resonances are in ±1 nm regime. The second tolerance, originated from primer density variability, has a more significant effect on the sensitivity. On top of the reported device, having a sensitivity of $742\frac{nm}{RIU}$ , we have measured additional 3 devices, as shown in Supplementary Figure S5, and obtained the following sensitivities: device #1 – $769\frac{nm}{RIU}$ , device #2 – $770\frac{nm}{RIU}$ , and device #3–762 $\frac{nm}{RIU}$ . From these results, we have found the variation in sensitivity is in the order of ∼10 $\frac{nm}{RIU}$ . The last tolerance can be further optimized in a future work. It should be noted that infected patients with SARS-CoV-2 were found to have a concentration [41] of 10^6^–10^11^ cp/mL, far beyond the fabrication tolerance of our device.

The results presented in this paper pave the way toward using MRRs for fast and accurate diagnosis of Covid-19. With minor modifications, this device can be tailored to detect different viral variants and even other pathogens. Moreover, it is possible to incorporate a stack of MRRs, each treated with a different primer. This way, our device can be further extended to detect a large variety of infectious conditions simultaneously. Furthermore, the level of integration can be enhanced by adding a microfluidic delivery system that will accurately control the delivery of analytes toward automization [42], as shown in Supplementary Figure S7.

Our platform can even detect a lower concentration of RNA, perhaps beyond the tens of cp/µL limit, by applying frequency lock stabilization techniques [43–45]. According to the reported literature [41], the SARS-Cov-2 molecule size is about 100 nm in diameter. Vollmer et al. reported a single virus detection based on whispering-gallery mode excited in a micro spherical cavity [46, 47]. He observed a change of 15 × 10^−8^ RIU spectral resonance shift for 45–55 nm size in radius particles with cavity Q factor of 2.6 × 10^6^. As a rule of thumb, one can expect that a single molecule of tens of nanometers in diameter can lead to a 10^−8^ refractive index change for resonators with diameters in the range of ours. Stern et al. [31] reported a detectable range of 5 pm in spectral difference between both resonances for Si double MRR integrated with frequency lock stabilization techniques with 5 × 10^4^ Q-factor. This value, which corresponds to ∼ 10^−8^ refractive index change, can be improved even further by changing the MRR’s material from Si to SiN, as reported in our work, due to the fact the thermo-optic coefficient is reduced by an order of magnitude from ∼10^−4^/C for Si to ∼10^−5^/C for SiN. It is worth mentioning that increasing the Q-factor may allow an even better noise floor. Therefore, we believe our platform holds the potential of detecting even lower changes in RIU, paving the way for single SARS-CoV-2 molecule detection.

## 3 Materials and methods

### 3.1 Device fabrication

Fabrication begins by depositing 400 nm of SiN layer onto 2.5 μm thick thermally grown silicon oxide. Following this, the photonic structure, including the bus waveguides and the MRRs (200 μm radii), is defined using standard electron-beam (e-beam) lithography, with 20 kV acceleration voltage and ZEP-520A as an e-beam resist (developed for 30 s). Next, the pattern is transferred to the SiN by inductively coupled reactive ion etching (ICP RIE) with a CHF_3_/O_2_ gas mixture for 250 s. The waveguides are designed to support a TE-like mode (transverse electric, in-plane polarization) with waveguide width and height of 400 nm and 1000 nm, respectively. Next, the upper cladding was constructed depositing 2 μm silicon oxide layer using PECVD. Finally, openings (square holes) were created in the top oxide layer above the MRRs using e-beam and etching process to allow the exposure of the MRR region to the analytes. An optical image of the MRRs can be seen in Supplementary Figure S6.

### 3.2 Probe MRR chemical functionalization

To functionalize the probe MRR, the PCR primer was chemically coupled to the sensor using surface chemistry. Briefly, the sensors were washed with ethanol absolute (Gadot group, Netanya, Israel) and dried using N_2_ flow. Then, the sensor surface was activated using O_2_ plasma treatment (Atto, Diener Electronic, Ebhausen, Germany) for 60 s. Next, the sensors were placed over a solution of 3-(aminopropyl) triethoxysilane (APTES) (Sigma-Aldrich, Jerusalem, Israel) in ethanol absolute with a concentration of 30 mM. The sensors and APTES solution were placed inside a vacuumed desiccator under an atmosphere of nitrogen gas for 2 h to allow the formation of a thin homogeneous layer [40, 48, 49]. The sensor was then dried at 75 °C for 10 min and cooled back to room temperature.

The free amine groups on the sensor were carboxylated by coupling with a dicarboxylic acid linker, glutaric acid (Sigma-Aldrich). The coupling was performed similarly to previously described procedure [50] with modifications. First, the glutaric acid was activated with N-(3-dimethylaminopropyl)-N′-ethylcarbodiimide hydrochloride (EDC) (Sigma-Aldrich) and N-hydroxysuccinimide (NHS) (Sigma-Aldrich). In a test tube, 8 mg glutaric acid were dissolved in 100 μL pure water (ultrapure deionized water, Milli-Q, Merck, Kenilworth, NJ) and mixed with 100 μL ethanol. Then 30 mg EDC and 15 mg NHS were dissolved in the glutaric acid solution and 200 μL 2-(N-morpholino)ethanesulfonic acid (MES) buffer solution (20 mM pH 6) were added. The glutaric acid coupling cocktail was mixed at 150 rpm for 20 min. Next, the glutaric acid coupling cocktail was titrated to pH 8 by titrating concentrated sodium hydroxide (5 M), and 25 μL were applied on the sensor probe MRR for 1 h. The sensor was thoroughly rinsed three times with ethanol:MES (1:1), then three times with pure water. To prevent the coupling cocktails from reaching the reference MRR, an epoxy barrier was applied between the two MRRs prior to the procedure. Hence, the glutaric acid linker and all subsequent chemical reactions were only performed on the probe MRR.

PCR primers functionalized with an amine group at the 5′ end (Sigma-Aldrich) were chemically coupled to the reactive NHS groups on the surface. We used the N1-R primer, with the sequence TCTGGTTACTGCCAGTTGAATCTG, designed by the CDC [36]. The primer coupling was performed by applying 25 μL primer solution 10 μM in carbonate buffer (50 mM pH 9) [50] to the sensor probe MRR. The primer solution was incubated over-night inside a box with a reservoir of pure water for humidity, to prevent evaporation of the primer solution. The sensors were then washed with three times with carbonate buffer, then three times with pure water, and dried under a flow of N_2_. The functionalized sensors were kept in a vacuumed desiccator until use.

### 3.3 Sensor surface characterization using scanning electron microscopy (SEM)

The surface of both MRRs and the bus waveguides was analyzed using HR-SEM and EDS (Sirion XL30 SFEG) and analyzed with internal EDS. Both MRRs were exposed to the same steps of chemical treatment, except the probe MRR was also exposed to the primer RNA. This fact was confirmed by EDS, which showed additional carbon atoms attached to the probe MRR surface. SEM and EDS figures are shown in Supplementary Figure S1.

We also compare those results to a clean SiN MRR without chemical activation to confirm that the additional carbon is originated from the activation process and is not present in the original sample.

### 3.4 Measurement details

The initial spectral frequencies of the resonances were measured prior to all measurements and used to calibrate the system. To characterize the fabricated device, we performed transmission measurements using a tunable laser operating at wavelengths around 1550 nm (HP 81608A), where the light is delivered into the chip using a lens fiber and coupled to the chip through an inverse tapered waveguide, using the edge (butt) coupling method. For thermal stabilization of the system, a temperature controller (Thorlabs TED2000) with TEC (TEC CUI devices cp3028) and digital thermistor (Thorlabs ad590) were used. Acetylene cell used to verify laser calibration (Thorlabs CQ09050-CH13 – Acetylene [^13^C_2_H_2_], 50 Torr).

### 3.5 Viral RNA sensing assay

Synthetic single-stranded RNA comprising SARS-CoV-2 RNA fragments [51] was used. A series of sequential 10-fold dilutions were made by diluting in RNAs free pure water. RNA solution at varying dilutions were applied in turn on the probe and reference MRRs, 10 μL each, and incubated for 7 min. During incubation, the resonance peak movement was monitored until steady state was achieved. After incubation, the sensor was carefully washed to remove any excess RNA molecules that adhered nonspecifically by dipping in RNAs free pure water three times for 1 min each and dried under N_2_ flow. The spectral difference between the probe and reference resonances were finally measured again after washing. Due to the sensor being reusable and not disposable, after each detection assay, the sensors were recycled. Recycling was performed by washing the sensor with boiling water. The double-strand melting temperature for the primers used in this study was calculated to be ∼54 °C [52]. Therefore, it is safe to assume that under boiling temperatures, there would be no primer-target RNA pairs left unbroken. Thus, this process unbinds the target RNA from the sensor, making the device ready for a new measurement. The spectral difference between the probe and reference resonances were back to the initial calibration resonances positions, which supports the assumption of reusability of the sensor. It is worth mentioning that the shift in signal over multiple measurement and recycling cycles should be tested thoroughly before such a device can be applied clinically. However, we did not observe a decrease in the device sensitivity over several testing cycles.

## 4 Summary

In summary, we have demonstrated a chip scale device consisting of a double MRR configuration for biphotonic applications. The MRRs consist of a SiN waveguide core and are designed to operate at the telecom spectral window around 1550 nm. Using this configuration, a sensitivity 743 nm/RIU was observed in the detection of SARS-COV-2 RNA molecules. While increasing the concentration beyond a certain value, saturation of the resonance shift was observed, probably due to the reduction in the number of active sites available for molecule bonding to the surface. For small concentrations, the spectral shift per additional RNA molecule is in the order of ∼1 pm. Thus, based on the reported sensitivity, the addition of a single molecule is expected to result in a change of ∼10^−6^ in RIU. Based on our previous work, this value is within the limits of detection [31]. As such, we believe that our approach can be optimized for the detection of molecules down to the single molecule level.

## Supplementary Material

Supplementary Material Details
