# Supplementary material for: On-chip multivariant COVID 19 photonic sensor based on silicon nitride double-microring resonators
Source: Nanophotonics. 2023 Mar 31;12(14):2831–9. doi: 10.1515/nanoph-2022-0722 (PMC11501435; doi:10.1515/nanoph-2022-0722)
Supplement: Supplementary file 1 — Supplementary Material Details [file j_nanoph-2022-0722_suppl_001.docx]

Supplementary material for “On-chip multi variant COVID 19 photonic sensor based on silicon nitride double-microring resonators”

Arieh Grosman^1,3^, Tal Duanis-Assaf ^2,3^, Noa Mazurski ^1,3^, Roy Zektzer^1,3^, Christian Frydendahl^1,3^ , Liron Stern^1,3^, Meital Reches^2,3^, and Uriel Levy^1,3^

*^1^ Department of Applied Physics, The Benin School of Engineering and Computer Science, The Hebrew University of Jerusalem, Jerusalem, Israel, 91904*

*^2^ The Institute of Chemistry, The Hebrew University of Jerusalem, Jerusalem, Israel, 91904*

*^3^ The Center for Nanoscience and Nanotechnology, The Hebrew University of Jerusalem, Jerusalem, Israel, 91904*

Figure S 1: EDS and HR SEM of the MRR’s surface. a- EDS of probe MRR, b- EDS of reference MRR. c,d – HRSEM of probe MRR in different scale.


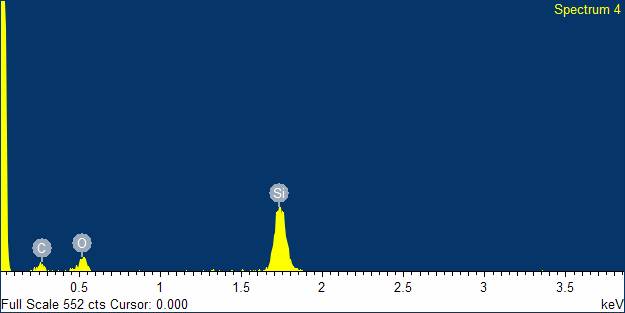

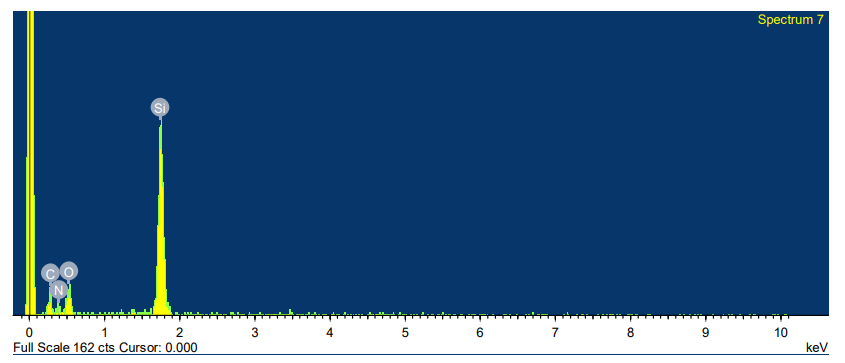

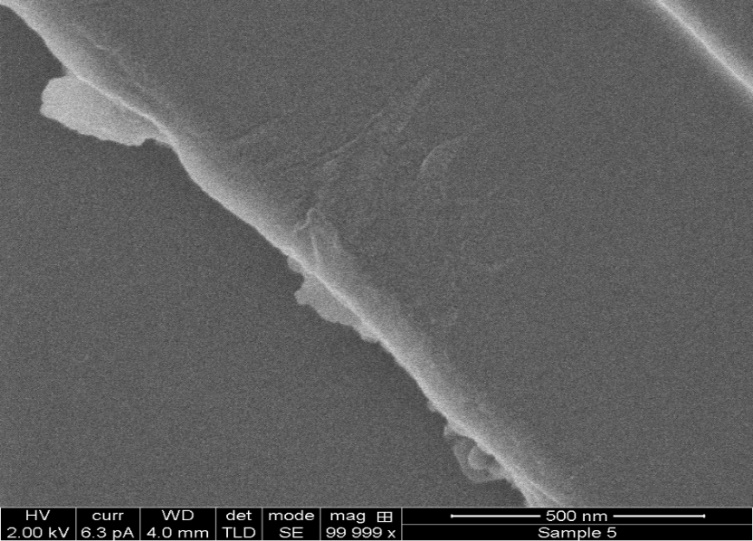

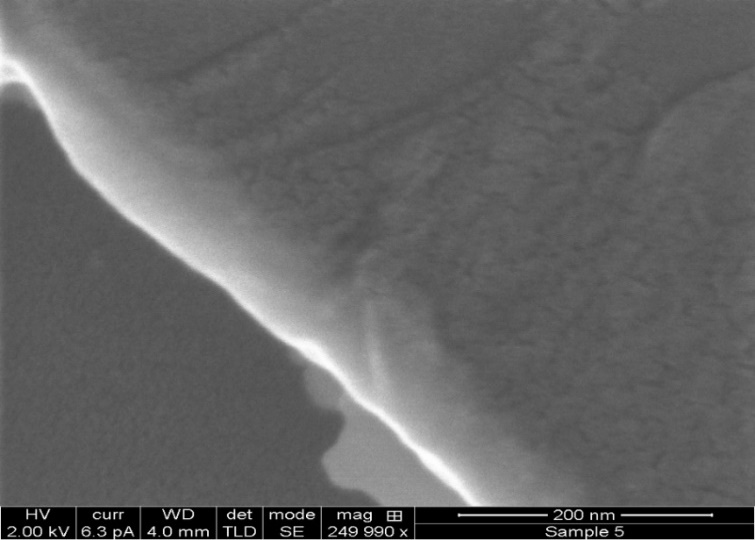


a

b

c

d

Figure S 2: Double-MRR resonance shift. Black – Resonance shift measured while applying RNA only on the probe MRR. Blue- drop the same RNA drop on both MRRs, no cleaning between measurements (starting measurements from the smallest amount).

Figure S 3: a small variance of sensitivity rises from different devices, coming from the tolerance of fabrication final dimensions and the density of the primer on the surface of the probe MRR.

Figure S 4: calibration measurement of 3 different devices (Device #1 -black line, Device #2 – red line, Device # 3 -blue line) as an outcome of lithography tolerance. the spectral distance ($\Delta$) between the reference and the probe resonances are about $\pm1 nm$ regime. The fabrication tolerance has a minor effect on the sensitivity due to the calibration process, where the shift originated from the additional RNA attached to the surface is compared with the initial measurement of the spectral distance between the two resonances.

Figure S 5: Measurement and linear fitting of the tolerance in primer density variability on the surface of the probe MRR from 3 different devices. The sensitivity was measured as: Device #1 (black line) $769\frac{nm}{RIU},$ device #2(red line) - $770\frac{nm}{RIU},$ and device #3 (blue line)- 762 nm/RIU. Considering also the device that was reported in the main text, having a sensitivity of $742\frac{nm}{RIU},$ the tolerance of the sensitivity was found to be ~ $10 nm/RIU$.

400 µm


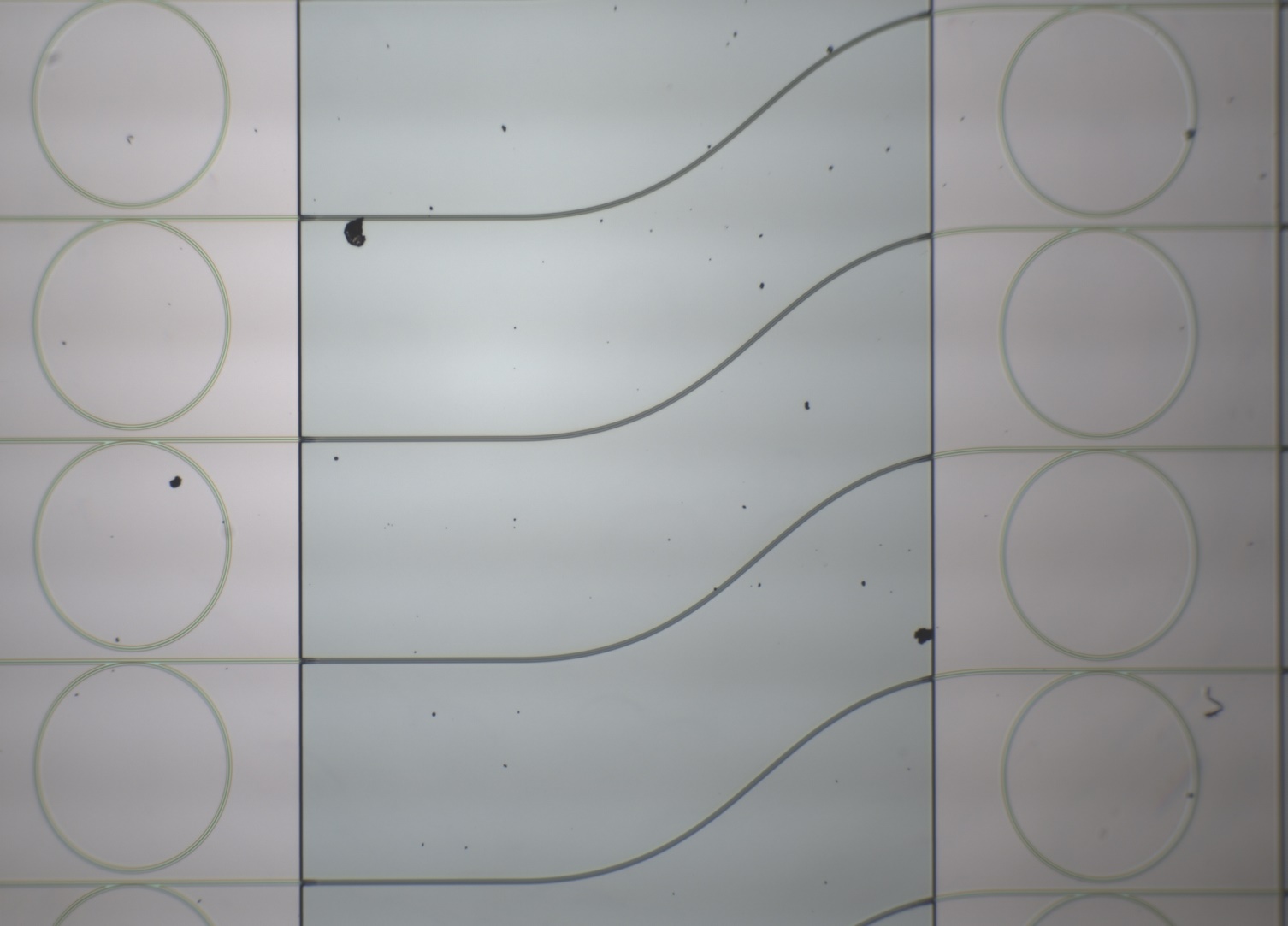


Figure S 6: Optical image of the double MRR. The radius of each MRR is 200µm, and the gap between waveguides and microring is 500 nm. the brighter color around the MRR shows the exposed oxide on top of the surface.


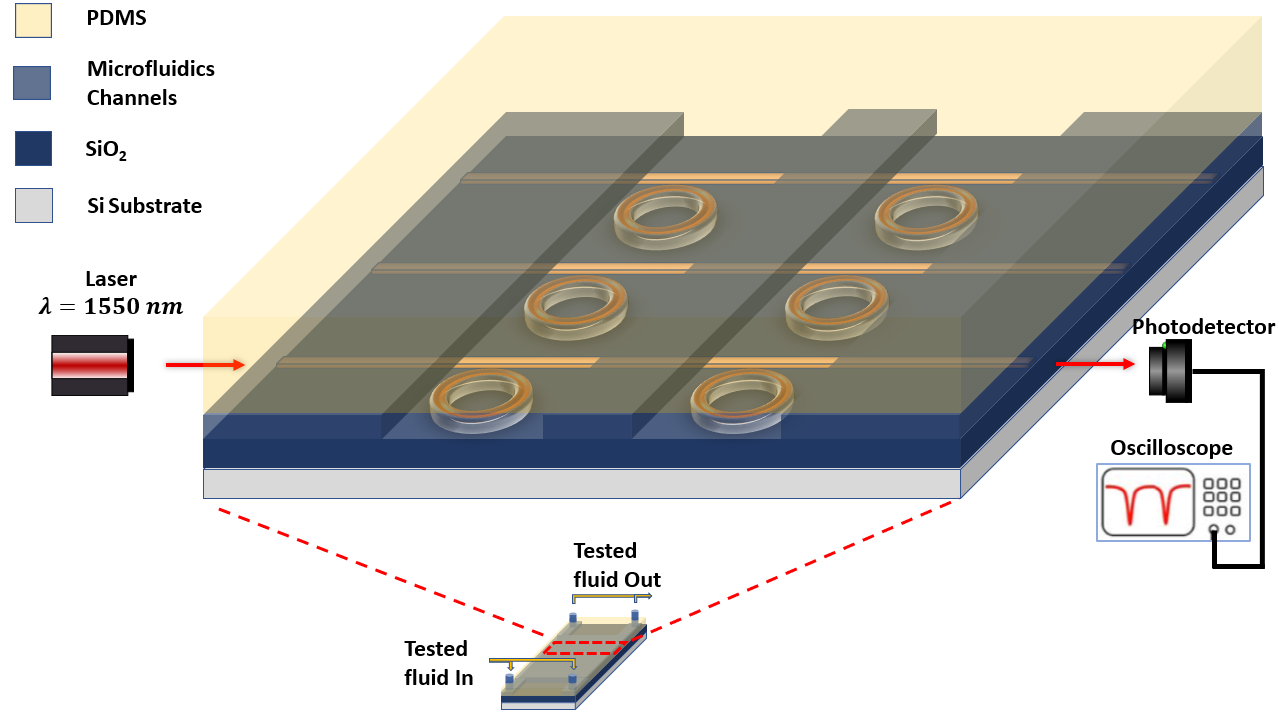


Figure S 7: System-level drawing of our vision for a multi-variant COVID-19 photonic sensing device. It consists of a stack of MRRs, each treated with a different primer. The top cladding is covered with PDMS microfluidics channels, that control the flow of liquids to be tested. This way, the device can be further extended to detect a large variety of infectious conditions simultaneously.
